# GPS tracking data of Lesser Black-backed Gulls and Herring Gulls breeding at the southern North Sea coast

**DOI:** 10.3897/zookeys.555.6173

**Published:** 2016-01-20

**Authors:** Eric W.M. Stienen, Peter Desmet, Bart Aelterman, Wouter Courtens, Simon Feys, Nicolas Vanermen, Hilbran Verstraete, Marc Van de Walle, Klaas Deneudt, Francisco Hernandez, Robin Houthoofdt, Bart Vanhoorne, Willem Bouten, Roland-Jan Buijs, Marwa M. Kavelaars, Wendt Müller, David Herman, Hans Matheve, Alejandro Sotillo, Luc Lens

**Affiliations:** 1 Research Institute for Nature and Forest (INBO), Kliniekstraat 25, 1070, Brussels, Belgium; 2 Flanders Marine Institute (VLIZ), Wandelaarkaai 7, 8400, Ostend, Belgium; 3 Institute for Biodiversity and Ecosystem Dynamics (IBED), University of Amsterdam, Science Park 904, 1098 XH, Amsterdam, The Netherlands; 4 Buijs Eco Consult B.V., Philips van Dorpstraat 49, 4698 RV, Oud-Vossemeer, The Netherlands; 5 Ethology (ETHO), University of Antwerp, Universiteitsplein 1, 2610, Antwerp, Belgium; 6 Terrestrial Ecology Unit (TEREC), Ghent University, K.L. Ledeganckstraat 35, 9000, Ghent, Belgium

**Keywords:** animal movement, bird tracking, GPS tracking, habitat use, migration, Lesser Black-backed Gull, Larus fuscus, Herring Gull, Larus argentatus, UvA-BiTS, LifeWatch, open data, MachineObservation, occurrence, observation

## Abstract

In this data paper, *Bird tracking - GPS tracking of Lesser Black-backed Gulls and Herring Gulls breeding at the southern North Sea coast* is described, a species occurrence dataset published by the Research Institute for Nature and Forest (INBO). The dataset (version 5.5) contains close to 2.5 million occurrences, recorded by 101 GPS trackers mounted on 75 Lesser Black-backed Gulls and 26 Herring Gulls breeding at the Belgian and Dutch coast. The trackers were developed by the University of Amsterdam Bird Tracking System (UvA-BiTS, http://www.uva-bits.nl). These automatically record and transmit bird movements, which allows us and others to study their habitat use and migration behaviour in great detail. Our bird tracking network is operational since 2013. It is funded for LifeWatch by the Hercules Foundation and maintained in collaboration with UvA-BiTS and the Flanders Marine Institute (VLIZ). The recorded data are periodically released in bulk as open data (http://dataset.inbo.be/bird-tracking-gull-occurrences), and are also accessible through CartoDB and the Global Biodiversity Information Facility (GBIF).

Research Institute for Nature and Forest

University of Amsterdam Bird Tracking System (

Flanders Marine Institute

Global Biodiversity Information Facility

## Data published through

http://doi.org/10.15468/02omly

## Rationale

As part of our terrestrial and marine observatory for LifeWatch (http://lifewatch.inbo.be), the Research Institute for Nature and Forest (INBO), the Flanders Marine Institute (VLIZ), Ghent University (UGent), and University of Antwerp (UA) are tracking large gull species with lightweight, solar powered GPS trackers. The project builds upon the extensive knowledge the INBO has acquired over the last 15 years when studying in particular postnuptial migration, as well as mate and site fidelity of large gulls, by means of sightings of colour-marked individuals ringed in Belgium and via individual-based life-history studies by UGent and UA. The data collected through this bird tracking network allows to study the migration patterns and habitat use of the gulls in more detail. Furthermore, they are no longer biased towards locations where observers can see colour-ringed birds. To allow greater use of the data beyond our research questions, all data are published as open data.

## Taxonomic coverage

**Figure 1.**
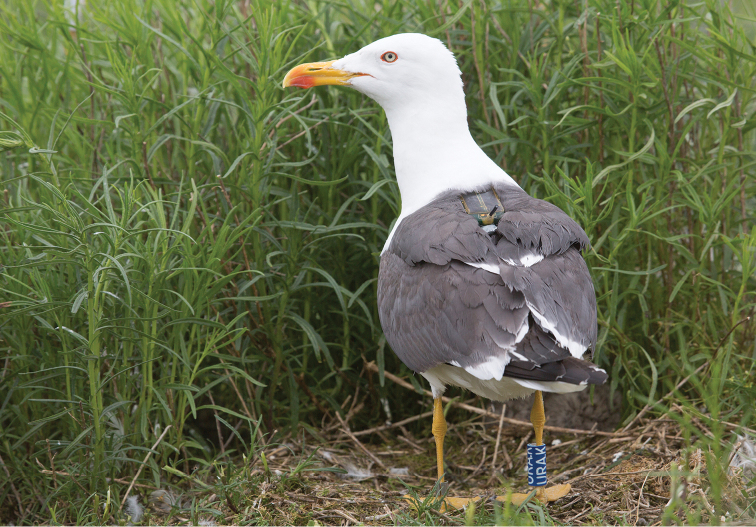
One of the tracked Lesser Black-backed Gulls (“Hans”, ring code: L906682), photographed near its nest in Zeebrugge on May 29, 2013 shortly after he was equipped with a tracker (device info serial: 861). Photo by Misjel Decleer, VLIZ photo gallery.

The dataset contains tracking data from 75 Lesser Black-Backed Gulls (*Larus fuscus*, Figure 1) and 26 Herring Gulls (*Larus argentatus*, Figure 3) breeding at the Belgian and Dutch coast.

### Taxonomic ranks

**Kingdom**: Animalia

**Phylum**: Chordata

**Class**: Aves

**Order**: Charadriiformes

**Family**: Laridae

**Genus**: *Larus*

**Species**: *Larus fuscus* (Lesser Black-backed Gull), *Larus argentatus* (Herring Gull)

## Geographic coverage

**Figure 2.**
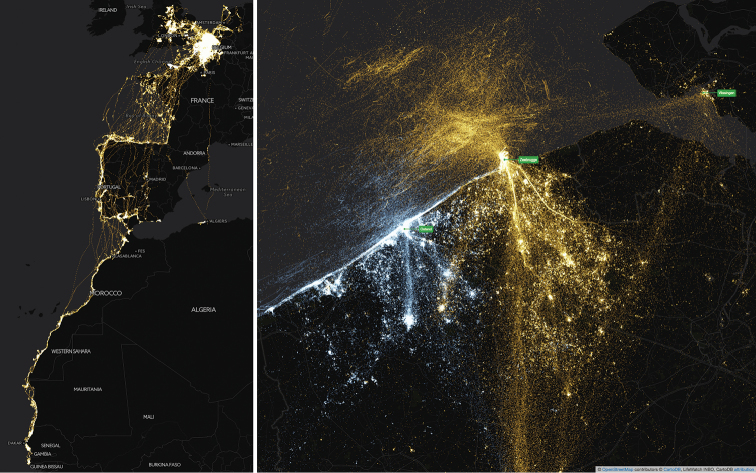
Left: map of western Europe and northwest Africa, showing the full extent of the gull tracking data, including two migration/wintering seasons. Right: map of the southern North Sea coast, showing mainly breeding season data. Each point represents a recorded occurrence, LBBG are indicated in orange, HG in blue. Overlapping points are brighter in colour. Maps created with CartoDB, basemap based on OpenStreetMap data. https://inbo.cartodb.com/u/lifewatch/viz/da04f120-ea70-11e4-a3f2-0e853d047bba/public_map

The tracked birds breed at the southern North Sea coast in three colonies, located in the ports of Zeebrugge (Belgium), Ostend (Belgium) and Vlissingen-Oost (the Netherlands). During the breeding season, their foraging range includes the west of Belgium and the Netherlands, northern France, the North Sea, and the English Channel. The Lesser Black-backed Gulls migrate south in winter, mainly hibernating in the south of Spain, Portugal, and North Africa (Figure 2).

### Bounding box

10° to 60° latitude, -25° to 10° longitude

## Temporal coverage

**Date range**: 2013-05-17 to 2015-09-02

**Formation periods**: breeding season 2013

**Formation periods**: migration/wintering season 2013-2014

**Formation periods**: breeding season 2014

**Formation periods**: migration/wintering season 2014-2015

**Formation periods**: breeding season 2015

## Methodology

### Study extent description

The birds were trapped and tagged at or near their breeding colony at the southern North Sea coast.

The colony of Zeebrugge is situated in the western part of the port (51.341 latitude, 3.182 longitude) at sites that are not used for port activities and on rooftops. The first Herring Gulls (HG) nested here in 1987, followed by the first breeding record of Lesser Black-backed Gull (LBBG) in 1991. In the 1990s, the number of breeding pairs strongly increased, with a maximum of 2,336 pairs of HG and 4,760 pairs of LBBG in 2011 (Stienen et al. 2015). Maximum numbers amounted to 2.6% and 1.2% of the biogeographic populations of LBBG and HG (Wetlands International 2015). After 2011 the number of gulls strongly declined due to habitat loss and the presence of foxes (*Vulpes vulpes*). In the period 2000–2010, Zeebrugge hosted on average 91% of all large gulls in Belgium. This proportion decreased to 33% in 2015 (Stienen et al. 2015).

In the colony of Ostend (51.233 latitude, 2.931 longitude), breeding started in 1993. Here the the numbers of breeding pairs are still increasing with a maximum of 505 pairs of HG and 551 pairs of LBBG in 2015 (data INBO). In Ostend most gulls breed on rooftops both in industrial areas and in the town itself.

The colony of Vlissingen-Oost also know as “Sloegebied” (51.450 latitude, 3.689 longitude) is located in the industrial port area near Vlissingen. Here the gulls nest on the grassy grounds that are not yet in use for port activities. LBBG started breeding in 1984, and the area is now the second biggest colony of LBBG in the southwestern part of the Netherlands. The numbers of breeding pairs increased from a few hundred in the second part of the nineties to 5,220 pairs in 2011. HG started breeding in 1977 (5–10 pairs) with a maximum of 4,353 pairs in 2008 (Strucker et al. 2013). In 2014 the colony hosted 4,460 pairs of LBBG and 2,276 pairs of HG (Strucker et al. 2015).

**Figure 3.**
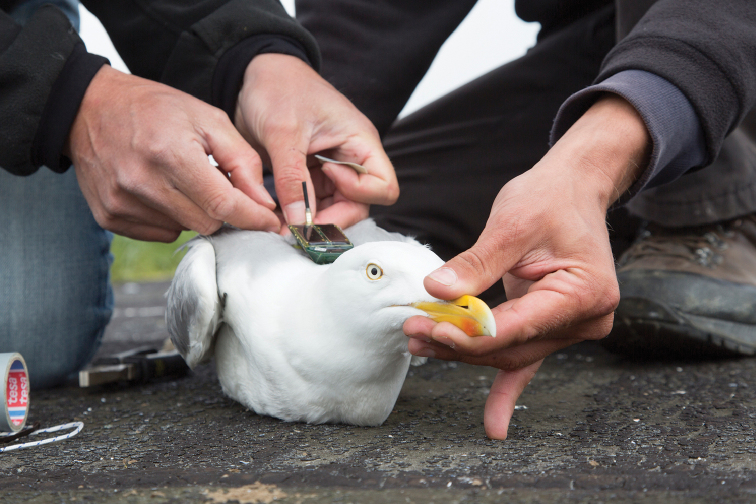
Researchers equipping a Herring Gull with a UvA-BiTS GPS tracker on the roof of the Vismijn in Ostend on May 24, 2013. Photo by Misjel Decleer, VLIZ photo gallery.

Most birds were trapped on their nest using a walk-in cage. We took biometrics of all captured gulls (bill length, bill depth, tarsus length, wing length, and body mass) and a feather sample to determine the sex. The UvA-BiTS GPS trackers were attached to the back of the gull using a harness of Teflon tape (Figure 3).

The number of tagged birds and their trap location per year are:

- 2013: 5 HG nesting on the roof of the Vismijn in Ostend and 22 LBBG nesting in the port of Zeebrugge.
- 2014: 8 HB nesting on the roof of the Vismijn in Ostend, 1 HG and 24 LBBG nesting in the port of Zeebrugge, and 3 HG feeding on the Visserskaai in Ostend (using a small cannon net).
- 2015: 9 HG nesting on the roof of the Vismijn in Ostend, 13 LBBG nesting in the port of Zeebrugge, and 16 LBBG nesting in Vlissingen-Oost.

### Sampling description

The birds are tracked with the University of Amsterdam Bird Tracking System (UvA-BiTS, http://www.uva-bits.nl). The system has been described in detail in Bouten et al. 2013. The lightweight, solar powered GPS trackers periodically record the 3D position and air temperature, and can be configured to collect body movements with the built-in tri-axial accelerometer as well. The system allows us to remotely set or change a measurement interval per tracker: the actual interval between measurements is provided in *samplingEffort* as *secondsSinceLastOccurrence*.

**Figure 4.**
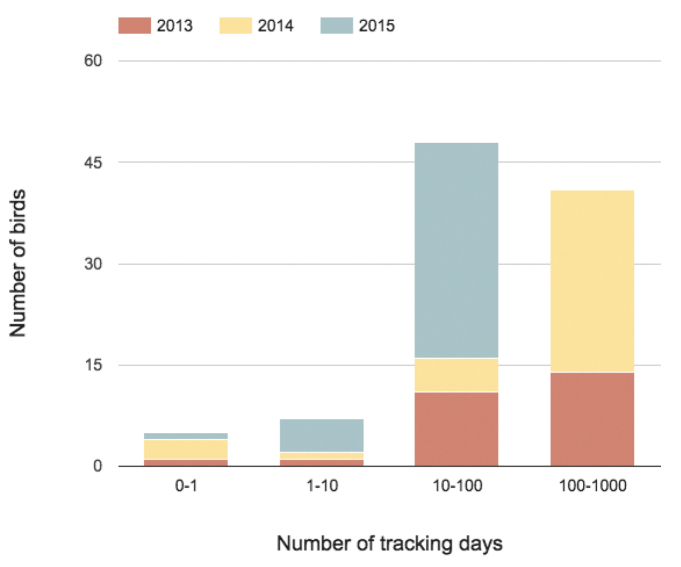
Number of birds grouped by number of tracking days and tracking start year.

The data are stored on the tracker, until these can be transmitted automatically and wireless to a base station using the built-in ZigBee transceiver with whip antenna. This receiver is also used to receive new measurement settings. The spatial range for this communication is restricted to the location of the base station (or antenna network), which is placed near the colony. Data cannot be retrieved from birds that do not return to the colony with the base station. For 3 of the 101 birds fitted with trackers no data were obtained and their *organismID*s (L909374, 5331094 ARNHEM, L909202) will thus not be found in the dataset. At the time of publication most individuals (88%) were tracked for more than 10 days and 41% for more than 100 days (Figure 4). The longest tracking period is 838 days (a HG with *organismID* H903185).

Data received by the base stations are automatically harvested, post-processed, and stored in a central PostgreSQL database at UvA-BiTS (http://www.uva-bits.nl/virtual-lab), which is accessible to the involved researchers only. We periodically export the tracking data to CartoDB for visualization purposes (see the External datasets section), removing test records and flagging outliers.

To create the Darwin Core Archive, we extract the data from the database and standardize these to Darwin Core using an SQL query (https://github.com/LifeWatchINBO/data-publication/blob/master/datasets/bird-tracking-gull-occurrences/mapping/dwc-occurrence.sql). The dataset is documented, published via our IPT (http://dataset.inbo.be/bird-tracking-gull-occurrences), and registered with the Global Biodiversity Information System (http://www.gbif.org/dataset/83e20573-f7dd-4852-9159-21566e1e691e). Issues or remarks regarding the data or this procedure can be reported at https://github.com/LifeWatchINBO/data-publication/tree/master/datasets/bird-tracking-gull-occurrences

To extract data from one individual, one can use *organismID*, which contains the unique metal leg ring code of each bird. Tracker IDs are provided in *dynamicProperties* as *device_info_serial*. For an overview of all GPS trackers and the individual birds these are mounted on, see https://inbo.cartodb.com/u/lifewatch/tables/bird_tracking_devices/public.

### Quality control description

See the section *Sampling description* for more details: our import procedure (https://github.com/LifeWatchINBO/bird-tracking/blob/master/cartodb/import-procedure.md) and standardization to Darwin Core (https://github.com/LifeWatchINBO/data-publication/blob/master/datasets/bird-tracking-gull-occurrences/mapping/dwc-occurrence.sql) are publicly documented.

### Method step description

1. Researcher captures bird, takes biometrics, attaches GPS tracker, and releases bird.
2. Researcher sets a measurement scheme, which can be updated anytime.
3. GPS tracker records data.
4. GPS tracker automatically receives new measurement settings and transmits recorded data when a connection can be established with the base station at the colony.
5. Recorded data are automatically harvested, post-processed, and stored in a central PostgreSQL database at UvA-BiTS.
6. LifeWatch INBO team periodically exports tracking data to CartoDB and makes these publicly accessible.
7. LifeWatch INBO team periodically (re)publishes data as a Darwin Core Archive, registered with GBIF.
8. Data stream stops when bird no longer returns to colony or if GPS tracker no longer functions (typical tracker lifespan: 2-3 years).

## Datasets

### Dataset description

- **Object name**: Bird tracking - GPS tracking of Lesser Black-backed Gulls and Herring Gulls breeding at the southern North Sea coast
- **Format name**: Darwin Core Archive format
- **Format version**: 1.0
- **Character encoding**: UTF-8
- **Language**: English
- **License**: http://creativecommons.org/publicdomain/zero/1.0/
- **Usage norms**: http://www.inbo.be/en/norms-for-data-use
- **Publication date**: 2014-06-18
- **Distribution**: http://dataset.inbo.be/bird-tracking-gull-occurrences
- **DOI**: http://doi.org/10.15468/02omly

### Usage norms

To allow anyone to use this dataset, we have released the data to the public domain under a Creative Commons Zero waiver (http://creativecommons.org/publicdomain/zero/1.0/). We would appreciate however, if you read and follow these norms for data use (http://www.inbo.be/en/norms-for-data-use) and provide a link to the original dataset (http://doi.org/10.15468/02omly) whenever possible. If you use these data for a scientific paper, please cite the dataset following the applicable citation norms and/or consider us for co-authorship. We are always interested to know how you have used or visualized the data, or to provide more information, so please contact us via the contact information provided in the metadata, opendata@inbo.be or https://twitter.com/LifeWatchINBO.

### External datasets

All our public bird tracking data are also available through CartoDB (https://inbo.cartodb.com/u/lifewatch), where users can query the data using SQL via the CartoDB API or download these in various formats (*csv*, *shp*, *kml*, *svg*, and *geosjon*). Two tables are of use: *bird_tracking*, containing all occurrence data and *bird_tracking_devices*, containing information on the GPS trackers and individual birds. Note that these tables are not standardized to Darwin Core, contain flagged outliers (omitted from the standardized dataset) and include data from other bird species. For more info, see https://github.com/LifeWatchINBO/bird-tracking/blob/master/cartodb/README.md

**bird_tracking**

- **Object name**: bird_tracking
- **Format name**: CartoDB table
- **Character encoding**: UTF-8
- **Distribution**: https://inbo.cartodb.com/u/lifewatch/tables/bird_tracking/public

**bird_tracking_devices**

- **Object name**: bird_tracking_devices
- **Format name**: CartoDB table
- **Character encoding**: UTF-8
- **Distribution**: https://inbo.cartodb.com/u/lifewatch/tables/bird_tracking_devices/public

### Additional information

The following information is not included in this dataset and available upon request: outliers, temperature, speed, accelerometer data, GPS metadata (fix time, number of satellites used, vertical accuracy), bird biometrics data measured during tagging (bill length, bill depth, tarsus length, wing length, body mass), life history data (day of ringing, age, resightings by volunteers), as well as growth data of chicks.

## Project data

### Project title

Bird tracking network

### Funding

This bird tracking network is funded for LifeWatch by the Hercules Foundation (http://www.herculesstichting.be/in_English/), with additional funding from the Research Foundation Flanders (FWO) to Wendt Müller and Luc Lens and Interreg Natura People (EFRO) through the Province of West Flanders.

### Project website

http://www.lifewatch.be/en/gps-tracking-network-large-birds
